# Frontostriatal functional connectivity in major depressive disorder

**DOI:** 10.1186/2045-5380-1-11

**Published:** 2011-12-08

**Authors:** Daniella J Furman, J Paul Hamilton, Ian H Gotlib

**Affiliations:** 1 Department of Psychology, Stanford University, 450 Serra Mall, Stanford, CA 94305-2004, USA

**Keywords:** depression, functional connectivity, striatum, subgenual anterior cingulate cortex, dorsolateral prefrontal cortex, fMRI

## Abstract

**Background:**

Abnormalities of the striatum and frontal cortex have been reported consistently in studies of neural structure and function in major depressive disorder (MDD). Despite speculation that compromised connectivity between these regions may underlie symptoms of MDD, little work has investigated the integrity of frontostriatal circuits in this disorder.

**Methods:**

Functional magnetic resonance images were acquired from 21 currently depressed and 19 never-disordered women during wakeful rest. Using four predefined striatal regions-of-interest, seed-to-whole brain correlations were computed and compared between groups.

**Results:**

Compared to controls, depressed participants exhibited attenuated functional connectivity between the ventral striatum and both ventromedial prefrontal cortex and subgenual anterior cingulate cortex. Depressed participants also exhibited stronger connectivity between the dorsal caudate and dorsal prefrontal cortex, which was positively correlated with severity of the disorder.

**Conclusions:**

Depressed individuals are characterized by aberrant connectivity in frontostriatal circuits that are posited to support affective and cognitive processing. Further research is required to examine more explicitly the link between patterns of disrupted connectivity and specific symptoms of depression, and the extent to which these patterns precede the onset of depression and normalize with recovery from depressive illness.

## Background

Major depressive disorder (MDD) is among the most prevalent and debilitating of all psychiatric illnesses, affecting nearly 20% of the US population, or more than 30 million adults, at some point in their lives [1]. MDD is characterized most commonly by sadness and/or by a marked decrease in the ability to experience reward or pleasure (that is, anhedonia). Individuals diagnosed with MDD also often exhibit psychomotor retardation and deficits in executive function, typically reflected by difficulties concentrating, making decisions, and terminating the processing of negative material [2]. Neuroanatomical models of depression, however, have yet to fully account for the range of symptom domains that characterize this disorder.

Investigators examining neural functioning in depressed individuals have frequently reported anomalies in the structure and function of the frontal lobes. For example, altered regional blood flow [3] and glucose metabolism [4] in the prefrontal cortex (PFC), a region frequently implicated in affect regulation and in a number of cognitive and motor processes, have been linked to both psychomotor retardation and anhedonia in depressed individuals. Interestingly, these studies also implicate anomalous functioning of the striatum in MDD, a revealing co-occurrence given the neuroanatomical linkages between the PFC and striatum. The striatum, composed within each hemisphere of the caudate nucleus, putamen, and core of the nucleus accumbens, comprises the primary input structure to the basal ganglia circuitry, receiving extensive projections from the frontal cortex. Output neurons from the basal ganglia send projections to midbrain structures and the thalamus, which completes a cortico-striatal-pallidal-thalamic (CSPT) circuit by returning projections to the frontal cortices. Whereas the PFC is generally thought to serve central control or goal representation functions, investigators have argued that CSPT circuitry aids in the filtering and focusing of cortical input, selecting among potential cognitive and behavioral representations [5,6]. This formulation has provided a foundation for models of behavior selection [6] and working memory updating [7], and supports the hypothesis that abnormal functioning of frontostriatal pathways contributes to the anhedonic, ruminative, and psychomotor dysfunctions characteristic of MDD.

Importantly, topographical organization within CSPT circuitry is largely preserved, such that corticostriatal neurons from the motor and premotor cortices project most densely to the caudal putamen ('motor' division), whereas input from dorsolateral PFC (DLPFC) innervates the dorsal caudate ('executive' division), and projections from the orbitofrontal cortex (OFC) and anterior cingulate cortex (ACC) tend to populate the ventral striatum ('limbic' or 'affective' division). In general, these divisions along the ventral-dorsal axis of the striatum are posited to be relatively segregated, thereby differentially supporting affective, cognitive, and motor processing [8]. Given that the difficulties experienced in MDD span these domains, it is possible that dysfunction in this system is attributable to global perturbations in microstructure or macrostructure, or in neurotransmission, rather than to abnormalities restricted to a particular CSPT division. Alternatively, the ascending flow of information through the parallel CSPT circuits [9] may enable a circumscribed abnormality in CSPT function to propagate to other subdivisions (for example, disrupting the translation of reward motivation into goal-directed motor behavior). Nonetheless, despite hypotheses that CSPT circuitry, and the frontostriatal connections in particular, may be compromised in MDD [10-12], little empirical work has explicitly examined CSPT circuit function in this disorder.

Over the last decade, there has been a surge in the development and use of methods to investigate network-level brain function [13]. These methods have recently been used to map the correlated activity, or functional connectivity, of normally and abnormally functioning corticostriatal networks in human subjects at rest [14,15]. Using one such method, investigators have documented attenuated functional connectivity between the ACC and a region of interest (ROI) spanning parts of the putamen and globus pallidus in depressed individuals [16]. This finding suggests anomalous frontostriatal connectivity in MDD, but does not permit us to draw conclusions about the subdivisions involved. In the present study, we examined patterns of frontostriatal connectivity in adults diagnosed with MDD, focusing on the dorsal and ventral striatal subdivisions posited to differentially support motor (dorsal putamen), executive (dorsal caudate), and affective (ventral caudate and putamen) functions, all of which have been found to be compromised in MDD. Given previous findings that mood [17] and reward-related behaviors [18] vary with the extent of connectivity between the ventral striatum (VS) and the subgenual anterior cingulate cortex (sACC), we hypothesized that, compared with non-depressed individuals, depressed participants would exhibit abnormal connectivity between the frontal cortex and the striatum, most markedly in this ventral, limbic circuit. Further, given the frequent presentation of cognitive and psychomotor dysfunction in MDD, we hypothesized that depressed persons would also exhibit attenuated frontostriatal connectivity within the executive and motor networks.

## Methods

### Participants

Twenty-one adults diagnosed with MDD and 19 control (CTL) participants with no history of psychiatric disorder participated in this study. Given the documented gender differences in the prevalence of depression [19], and to reduce possible heterogeneity in neural functioning, we included only female participants in our sample. Participants were recruited from local psychiatric outpatient clinics and through advertisements posted in numerous locations within the community. Participants' responses to a telephone interview provided initial selection information. This phone screen established that participants were fluent in English and were between 18 and 60 years of age. Participants were excluded if they reported severe head trauma, learning disabilities, or bipolar disorder. Participants were also excluded if they met criteria for alcohol or substance dependence or for alcohol or substance abuse within the past 6 months. Trained interviewers administered the Structured Clinical Interview for *Diagnostic and Statistical Manual of Mental Disorders, fourth edition *(DSM-IV) Axis I Disorders (SCID) to eligible individuals; in previous studies, our team of interviewers has demonstrated excellent inter-rater reliability for both the diagnosis of MDD (κ = 0.93) and for the non-psychiatric CTL diagnosis (κ = 0.92). All depressed participants met criteria for a DSM-IV diagnosis of current MDD based on their responses to the SCID. None of the control participants met diagnostic criteria for any current or past Axis-I disorder. In addition, all participants completed the Beck Depression Inventory II (BDI) [20], a self-report measure of the severity of depressive symptoms, and the 22-item Ruminative Responses Scale (RRS) of the Response Styles Questionnaire [21], a self-report measure of ruminative tendencies. Informed consent was obtained from all participants, and all aspects of this study complied with the ethical standards for treatment of human participants from the American Psychiatric Association.

### fMRI data acquisition

Blood-oxygen-level dependent (BOLD) signal data were acquired with 3 Tesla General Electric magnetic resonance imaging scanners (General Electric, Milwaukee, WI, USA) using single-channel whole-head coils. Images were acquired used a T2*-weighted, in/out spiral pulse sequence [22]. Scans were acquired as part of three different imaging studies and, therefore, had slightly different parameter specifications (version A: repetition time (TR) = 1,200 ms/frame, echo time (TE) = 30 ms, flip angle = 77, field of view (FOV) = 220 mm, number of temporal frames (NTF) = 250, 18 slices, 3.44 mm^2 ^in-plane and 5 mm through-plane resolution; version B: TR = 1,300 ms/frame, TE = 30 ms, flip angle = 80, FOV = 220 mm, NTF = 190, 20 slices, 3.44 mm^2 ^in-plane and 5 mm through-plane resolution; version C: TR = 2,000 ms/frame, TE = 30 ms, flip angle = 77, FOV = 220 mm, NTF = 180, 29 slices, 3.44 mm^2 ^in-plane and 4 mm through-plane resolution). High-resolution anatomical scans were collected for every participant using a spoiled grass gradient recalled (SPGR) sequence for T1 contrast (TE = 7 ms, flip angle = 15, FOV = 220 mm, 124 axial slices, 0.86 mm^2 ^in-plane and 1.2 mm through-plane resolution). Participants were instructed to lie still during the scan with their eyes closed.

### Data preprocessing

All data preprocessing and analysis were carried out using Analysis of Functional NeuroImages (AFNI; http://afni.nimh.nih.gov/afni/) software. The following procedures were applied to all datasets regardless of data acquisition parameters: (1) removal of first three brain volumes to allow for equilibration of longitudinal magnetization; (2) slice-time correction to the middle axial slice; (3) coregistration to the middle temporal acquisition to correct for head rotation and translation during the scan (Fourier interpolation); (4) temporal band-pass filtering at 0.009 Hz <*f *< 0.08 Hz, (5) spatial smoothing with a 6 mm Gaussian smoothing kernel; (6) conversion to units of percent signal change; (7) spatial resampling to 27 mm^3 ^isotropic voxels; and (8) coregistration to high-resolution anatomical images and affine transformation to Talairach space.

Previous investigators have demonstrated the feasibility of pooling data with varying acquisition parameters from multiple studies [23]. Nevertheless, to maximize homogeneity across the three scan types, after preprocessing we took several additional steps to correct for the discrepancy in temporal sampling rate. First, we upsampled all voxel time series data from scan versions A and B using linear interpolation by a factor necessary for subsequent resampling to TR = 2,000 ms/frame (i.e., 6 for version A, 13 for version B). We then downsampled data by the required amount (i.e., selection of every 10th data point for version A and every 20th point for version B). Finally, we resampled head motion data in the same manner as corresponding time-series data. Limiting our analyses to the duration of the shortest scanning session (B), we used only the first 122 time points of each preprocessed dataset, resulting in an effective scan duration of 4 minutes, 4 seconds.

### Nuisance signal removal and ROI time series extraction

Consistent with previous studies of basal ganglia functional connectivity [14,24], we regressed participants' preprocessed data on a series of noise predictors, modeling the zero-order through second-order trends in the BOLD time series, and nuisance signals from white matter (WM), cerebrospinal fluid (CSF), global signal, and six motion parameters (three rotational and three translational). Resulting terms were used to generate time series for regressors of no interest, which were then subtracted from the original preprocessed data [25].

For purposes of ROI seed placement, we used eight striatal seeds (four per hemisphere) described by Di Martino *et al*. [14] converted from Montreal Neurological Institute (MNI) space into Talairach coordinates by means of a non-linear transform [26]. In each hemisphere, seed ROIs comprised a spherical mask with 7 mm radius centered in ventral striatum (VS; Talairach × y z: ± 8.9 8.4 -7.1), dorsal caudate (DC; ± 12.9 14.9 7.5), dorsal caudal putamen (DCP; ± 27.7 1.1 2.7), and ventral rostral putamen (VRP, ± 19.8 11.5 -3.1). As detailed by Di Martino *et al*. [14], the locations of these seeds are consistent with identified subdivisions of the striatum. For each participant, the locations of seeds were visually inspected with reference to anatomical images to ensure appropriate placement. BOLD time-series data averaged across all voxels within each of the eight ROIs were then extracted from participants' noise-covariate corrected data. Finally, time-series data from corresponding right and left hemisphere ROIs were averaged to form four bilateral ROIs.

### Statistical analyses

We regressed each participant's preprocessed voxel time series data separately for each of the four bilateral seed regions on the spatially averaged ROI time series along with the nuisance covariates (zero-order through second-order trends in the BOLD time series, six motion parameters, and signal from WM, CSF, and whole-brain masks). By regressing the preprocessed data rather than the residuals at this stage, we were able to orthogonalize the basal ganglia seeds with respect to the nuisance covariates, thereby ensuring that the resulting statistical maps reflected the unique contribution of seed mask time series. These analyses yielded subject-level t-statistic maps indexing regional correlations with the ROI time series. Individual statistical maps were Fisher transformed to Z-score maps prior to analysis at the group level. To compare patterns of correlated activity with each bilateral seed region between MDD and CTL samples, we conducted two-sample t-tests on participants' Z-score maps. To elucidate the nature of the group differences, we conducted one-sample t-tests on patterns of striatal functional connectivity within each group. We conducted 2,000 Monte Carlo simulations using AFNI's AlphaSim [27] and determined that an uncorrected single voxel significant threshold of *p *< 0.005 and cluster threshold of *k *= 26 were necessary to hold family-wise type I error at *P *< 0.05.

### Exploratory analyses

For each suprathreshold frontal cortical cluster that was identified in the between-groups analyses, connectivity or correlation coefficients (r) were averaged across all voxels falling within the cluster. Within our sample of MDD participants, we then correlated averaged estimates of connectivity strength with measures of disorder severity (BDI) and rumination (RRS). In addition, we conducted two-sample t-tests comparing average correlation coefficients from these clusters between those MDD participants with and without observable psychomotor symptoms (determined on the basis of SCID criteria). Given the exploratory nature of these analyses, we used an uncorrected statistical threshold of *p *< 0.05 for each test.

## Results

### Demographic and clinical characteristics

The MDD and CTL groups did not differ significantly in age (MDD: mean = 39.2, SD = 11.8; CTL: mean = 33.2, SD = 10.5; *t*(38) = 1.7, *p *> 0.1) or in the distribution of data acquisition protocols from which participants were drawn (χ^2^(2) = 1.24, *p *> 0.5). (Age did not moderate levels of functional connectivity for any of the frontal cortical regions reported in the between-group analyses. In addition, we did not observe effects of acquisition protocol, or of an acquisition protocol-by-group interaction, on estimates of frontostriatal connectivity in regions reported here to vary by group.) As expected, scores on the BDI were significantly higher for the MDD (mean = 31.3, SD = 11.7) than for the CTL (mean = 1.5, SD = 2.4) participants, *t*(38) = 10.9, *p *< 0.0001. Fourteen of the participants with MDD were taking at least one of the following psychotropic medications: venlafaxine, bupropion, escitalopram, paroxetine, fluoxetine, duloxetine, atomoxetine, mirtazapine, trazadone, buspirone, temazepam, zoldipem, modafinil, and buprenorphine. (Psychoactive medication status, defined as a dichotomous variable, did not significantly mediate any of the reported frontostriatal connectivity effects [see Additional file 1 for comparisons].) In addition, nine of the MDD participants met diagnostic criteria for at least one comorbid Axis-I disorder: dysthymia (n = 1); panic disorder (n = 2); post-traumatic stress disorder (PTSD) (n = 2); anorexia (n = 1); and social anxiety disorder (n = 5). One CTL participant and two MDD participants reported being regular smokers.

### Functional connectivity

**Figure 1:**
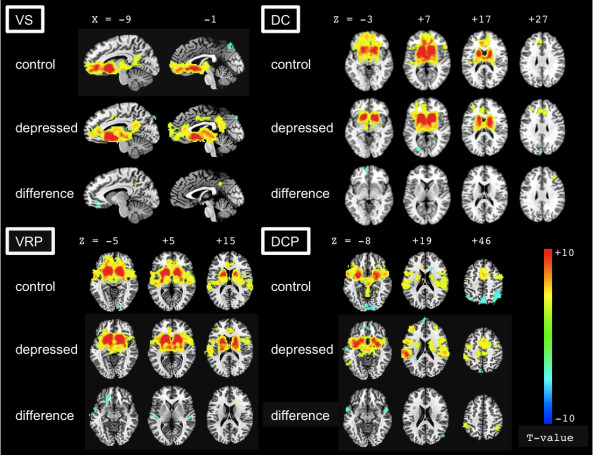
**Statistical maps depicting regions of positive and negative functional connectivity with the striatum**. Statistical maps depicting regions of positive and negative functional connectivity with the ventral striatum (VS), dorsal caudate (DC), ventral rostral putamen (VRP), and dorsal caudal putamen (DCP) in depressed and control samples, and between-group differences in functional connectivity with these regions (blue = control > depressed; yellow = depressed > control). Voxel-wise *p *< 0.005, minimum cluster size = 26 voxels.

The patterns of functional correlations with caudate and putamen identified in the samples of MDD and CTL participants were, in general, consistent with those reported by Di Martino *et al*. [14] (Figure 1 [see Additional files 2 and 3]). Briefly, in CTL participants, activity in the VS was correlated with activity in an extensive region of bilateral caudate, ventromedial prefrontal cortex (vmPFC), OFC, sACC, and posterior cingulate cortex (PCC), as well as with areas of the hippocampal formation, insula, superior temporal gyrus, medial dorsal thalamus, and brainstem. Activity in the DC seed was correlated with activity in regions of dorsal ACC and PFC, medial dorsal thalamus, putamen, inferior frontal gyrus, and the cerebellum.

As in the VS, activity in VRP seeds was correlated with a large region extending through the putamen, ventral caudate, superior temporal gyrus, insula, globus pallidus, and vmPFC. Activity in these seeds was also correlated with activity in the precentral gyrus, cerebellum, and a region of dorsomedial PFC bridging the dorsal aspect of the ACC and supplementary motor area (SMA). The DCP seeds were functionally connected to several cortical and subcortical regions including the globus pallidus, insula, inferior frontal and superior temporal gyri, thalamus, cerebellum, and a region of dorsomedial PFC abutting ACC and SMA. Finally, activity in the right DCP was correlated with activity in the precentral and postcentral gyri.

Given our specific hypotheses of altered frontostriatal connectivity in MDD between striatal seed regions and the frontal cortex, we report significant differences in frontostriatal connectivity between the MDD and CTL participants for each seed region (Figure 1). Detailed results of these analyses, as well as group differences in regions outside of the frontal cortex, are presented in Table 1.

**Table 1:** Between-group differences in striatal connectivity

Seed	Contrast	Region	Peak voxel (Talairach)			Cluster size	Peak t value
					
			x	y	z		
VS	CTL > MDD	Left subgenual ACC, medial orbitofrontal cortex	-13	41	-4	40	3.9
	MDD > CTL	Left precuneus, posterior cingulate cortex	-7	-46	32	39	4.1
		Right cerebellum	29	-25	-28	31	4.4
		Right inferior parietal lobule	35	-49	38	30	3.9
DC	CTL > MDD	Left medial frontal gyrus	-7	53	-1	32	3.6
	MDD > CTL	Right middle frontal gyrus	32	32	26	44	4.0
VRP	CTL > MDD	Left superior temporal gyrus	-58	-13	-1	93	4.2
		Left medial frontal gyrus, orbitofrontal cortex	-16	35	-10	78	4.5
		Right posterior insula	44	-13	8	30	4.0
	MDD > CTL	Right caudate body, subgyral	17	14	20	52	4.4
DCP	CTL > MDD	Left superior temporal gyrus	-55	2	-7	50	5.1
		Right superior temporal gyrus	53	5	-10	46	4.6
		Right posterior insula	41	-16	11	44	4.4
		Right middle temporal gyrus	53	-64	14	41	3.8
	MDD > CTL	Left inferior parietal lobule, postcentral gyrus	-46	-37	50	80	4.8
		Right inferior parietal lobule	41	-46	47	54	4.7
		Right lingual gyrus	26	-85	-13	38	4.4

Voxel-wise *p *< 0.005, minimum cluster size = 26 voxels.

ACC = anterior cingulate cortex; CTL = control; DC = dorsal caudate; DCP = dorsal caudal putamen; MDD = major depressive disorder; VRP = ventral rostral putamen; VS = ventral striatum.

#### Ventral striatum

Consistent with our hypotheses, we observed attenuated functional connectivity between VS and both vmPFC and sACC in MDD, relative to CTL, participants.

#### Dorsal caudate

The MDD participants exhibited decreased functional connectivity between the DC seed and the medial frontal gyrus, relative to CTL participants. MDD participants also showed increased positive connectivity between the DC and DLPFC.

#### Ventral putamen

Compared with CTL participants, individuals with MDD exhibited diminished connectivity between the VRP and ventromedial regions of the frontal lobe.

#### Dorsal putamen

There were no significant differences between MDD and CTL participants in frontal-DCP connectivity.

### Associations with clinical variables

**Figure 2:**
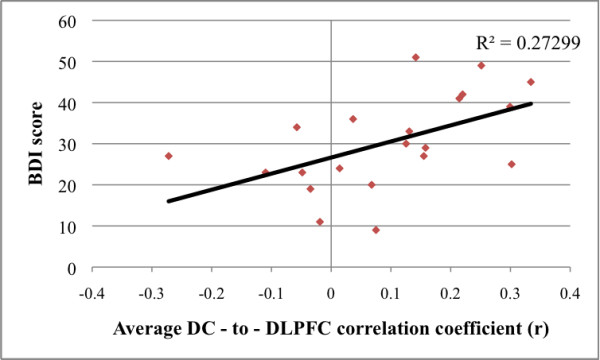
**Relation between depression severity and dorsal caudate (DC) to dorsolateral prefrontal cortex (DLPFC) connectivity**. Relation between depression severity and dorsal caudate (DC) to dorsolateral prefrontal cortex (DLPFC) functional connectivity in currently depressed participants. BDI = Beck Depression Inventory II.

RRS data were collected from all but two participants. BDI and RRS scores were moderately correlated, r = 0.47, *p *< 0.05. Of the four frontostriatal relations examined (VS-sACC, DC-medial frontal gyrus, DC-DLPFC, VRP-vmPFC), only DC-DLPFC connectivity was significantly correlated with depression severity (n = 21, r = 0.52, *p *= 0.015; see Figure 2); for all other correlations with BDI, |r| < 0.30, *p*s > 0.2. Although none of the connectivity measures was significantly correlated with RRS score, there was a trend for DC-DLPFC connectivity to correlate with RRS score (n = 19, r = 0.38, *p *= 0.105); for all other correlations with RRS, |r| < 0.18, *p*s > 0.4. Depressed participants with observable psychomotor retardation (n = 11) exhibited significantly weaker functional connectivity between the DC and the medial frontal gyrus (mean = 0.003, SD = 0.20) than did depressed participants without this symptom (n = 10; mean = 0.162, SD = 0.14), t(19) = 2.1, *p *= 0.05. There was a trend for depressed participants with psychomotor retardation to exhibit weaker VRP-vmPFC connectivity, and stronger DC-DLPFC connectivity, than did depressed participants without psychomotor symptoms, but these differences were not statistically significant, all t(19) < 1.2, *p*s > 0.17.

## Discussion

The present study was designed to examine alterations in the functional connectivity of frontostriatal networks in MDD. Patterns of connectivity in both control and depressed participants were consistent with the results of previous studies [14]; importantly, however, striking differences between the depressed and non-depressed participants were detected in networks that have been implicated in affective and motivational processing [28]. In particular, we found that, compared with healthy controls, depressed individuals exhibited attenuated connectivity between seed regions approximating the ventral striatum/nucleus accumbens and ventral rostral putamen, and vmPFC and sACC targets.

It is important to note that these prefrontal target regions fall within the bounds of regions known to have anatomical connectivity with the VS [8,9]. As noted earlier, the pattern of projections from the cortex to the striatum maintains much of its anatomical distinction, such that corticostriatal neurons from the motor and premotor cortices project most densely to the caudal putamen, whereas input from the OFC and vmPFC tends to populate the anteroventral caudate and ventromedial putamen. Indeed, this ventral corticostriatal network is posited to support reward-related processing and affect [8]. Consistent with this framework, several of the ventromedial PFC structures found in this study to correlate differentially with striatal activity in participants with MDD and CTL subjects have previously been implicated in depression and in the processes affected by this disorder [29,30].

Because of its unique interconnectivity with subcortical and limbic structures, the sACC has been hypothesized to be involved in both affective processes and visceromotor integration [31,32], and has specifically been associated with the induction of negative mood [33]. Functional [29], metabolic [34], and volumetric [34,35] anomalies involving the sACC are often reported in studies of MDD. Interestingly, Harrison and colleagues [17] reported a relation between cytokine-induced decrements in mood and the degree of connectivity between the nucleus accumbens and the sACC. Considered in light of our current findings, this body of work suggests that multiple forms of brain pathology, including aberrant structure or functionality of either striatal or frontal regions, ultimately produce a similar, deleterious effect on network-level function. Thus, future research should examine the extent to which volumetric and metabolic anomalies contribute to the pathophysiology of MDD by altering patterns of mesolimbic or corticostriatal connectivity.

Situated just rostral to the sACC, the vmPFC is associated with the production [36] and flexible shifting of emotional states [37] and behavior, as well as with reward processing [38] and reinforcement learning [39]. Interestingly, the integrity of fibers linking the vmPFC, as well as the medial OFC, with the VS appears to mediate individual differences in reward-related behavior. For example, using diffusion tractography, Cohen and colleagues found a positive correlation between the integrity of this tract and reward dependence, a personality trait characterized by learning from reward signals and persistence in repeating actions associated with rewards [18]. Importantly, impaired reinforcement or reward learning is characteristic of MDD, and has been linked to anhedonic symptoms in depressed individuals [30]; the current finding of diminished functional connectivity between the vmPFC and VS in MDD suggests a potential neural correlate of this deficiency. Future work should examine the relation between differences in frontostriatal connectivity and compromised motivational and reward-learning processes in anhedonic depression.

We also observed attenuated connectivity between the dorsal striatum and medial PFC in depressed, relative to control, participants. In contrast to the ventral corticostriatal 'limbic' networks, those networks comprising more dorsal (and caudal) regions of the striatum, along with their respective inputs from the frontal cortices, are thought to support executive function and motor-planning processes, including the initiation of behavior [40]. In addition to detailing associations between MDD and compromised executive and motor function, studies have linked depression to difficulties in the initiation of motor movements [41]. For example, Sabbe *et al*. report that the highest degree of motor slowing in MDD is observed during the early stages of a movement, suggesting a particular difficulty in initiating behaviors [42]. Subsequent work has demonstrated an increased reliance on external cues during the performance of sequential movements in melancholic depressed persons, reminiscent of the deficits of initiative seen in Parkinson's disease (PD) [43]. While preliminary analysis of the relation between psychomotor retardation and dorsal striatal connectivity suggests a plausible mechanism by which psychomotor function is compromised in MDD, future research with larger sample sizes and more detailed assessments of symptom severity will help clarify the extent and specificity of this relation.

We found an unanticipated increase in connectivity between the dorsal caudate and the middle frontal gyrus in MDD. Given the association between dorsal corticostriatal macrocircuitry and both the filtering and manipulation of content in working memory [44,45], it is possible that this result reflects the inability to update the contents of working memory [46] and the maintenance of negative information in MDD [47]. Preliminary support for this formulation comes from our observation that, for depressed participants, increasing tendency to ruminate predicted increasing DC-DLPFC connectivity during rest, though this correlation did not reach statistical significance in the current sample. It is possible that the observed increase in functional connectivity reflects a compensatory mechanism; if output from the basal ganglia circuitry to the frontal cortex (via the thalamus) is compromised in individuals with depression, cortical input to the basal ganglia may be ramped up in an attempt to drive an underactive basal ganglia-thalamus complex.

In addition to differences between depressed and non-depressed participants in the degree or extent of connectivity within corticostriatal networks, alterations observed in this study in the relation between these networks and other known brain systems are noteworthy. Relative to healthy individuals, participants with MDD exhibited enhanced connectivity between the VS and the PCC, a cortical-midline region central to the default mode network (DMN) [48]. The DMN, composed of medial frontal, lateral and medial parietal, as well as temporal lobe regions, has been implicated broadly in self-referential processing [49]. Activity across the various nodes of this network is reduced during periods of non-self-referential processing, such as those that occur during the performance of demanding cognitive tasks [48,49]. Recently, Kelly *et al*. demonstrated that administering L-3,4-dihydroxyphenylalanine (L-DOPA), a precursor to dopamine (DA), to healthy participants diminishes functional connectivity between the PCC and caudate regions [24]; these authors propose that the striatum plays a role in the suppression of the DMN under conditions requiring a shift from self-directed, to externally-directed, processing. In contrast, our finding indicates a *reduction *in the anticorrelation of these two networks in MDD, potentially reflecting the difficulty exhibited by depressed persons shifting attention away from self-referential processing and reducing DMN activity in the face of external demands [50].

Although we did not quantify dopaminergic factors in the present study, there is reason to believe that DA plays an important role in the modulation of these corticostriatal connections. Whereas augmentation of DA levels has been shown to increase functional connectivity between the VS and vmPFC [24], DA depletion has been found to attenuate the functional coupling between VS and both dorsal and ventral PFC [51]. Similarly, connectivity analyses in PD patients have demonstrated attenuated connectivity between the ventromedial caudate (approximating our VS seed) and the vmPFC [52]. The similarity between the connectivity patterns identified in both temporary and disease-related DA-depleted states and those identified in our depressed sample is intriguing, and argues for a more systematic investigation of the relations among DA tone, corticostriatal connectivity, and symptom presentation in MDD.

In closing, we note a number of limitations of this study. First, our sample was composed entirely of females, thereby limiting our ability to generalize the current results to male subjects. Second, our depressed sample was heterogeneous with respect to comorbid DSM-IV diagnoses and psychoactive medication status. Although statistical tests indicated that medication status [see Additional file 1] did not mediate the aberrant patterns of frontostriatal connectivity we found to characterize depressed individuals, we cannot rule out the possibility that certain drug classes might selectively affect these circuits. While previous studies have reported no change in resting state basal ganglia-ACC functional connectivity following selective serotonin reuptake inhibitor (SSRI) treatment [53] and only decreases in DC-frontal lobe connectivity following administration of L-DOPA to healthy controls [24], future research might examine directly the effects of dopaminergic agents on corticostriatal connectivity, particularly in the context of symptom remediation. Third, we may be underpowered in the current study to detect and precisely characterize the associations between clinical variables and frontostriatal abnormalities. It is also possible that certain of the documented abnormalities in frontostriatal connectivity reflect risk factors for the development of depression. In this case, we might not expect to identify correlations with disease or symptom severity. These possibilities highlight the importance of examining corticostriatal connectivity in a larger sample of MDD subjects, in those at elevated risk for the development of MDD, and in individuals who have recovered from the disorder. Finally, the present findings of altered frontostriatal connectivity in MDD may be attributable, in part, to structural changes within the striatum itself [54-56]. Although we assume that the seeds used in this study represent discrete functional divisions of the striatum, it is possible that the boundaries between such divisions are shifted in the brains of depressed persons as a result of structural alteration. Future work directly examining the consequences of gross anatomical, metabolic and/or functional changes on frontostriatal circuitry could advance our understanding of how regional changes in brain function alter system-level function in psychiatric disorders. Despite these limitations, however, the results of the present study provide important empirical support for the hypothesis that MDD is characterized, and even maintained, by compromised frontostriatal function.

## Competing interests

The authors declare that they have no competing interests.

## Authors' contributions

DJF and IHG designed the study, and DJF and JPH conducted the statistical analyses. DJF wrote the first draft of the manuscript. All authors contributed to, and approved, the final manuscript.

## Supplementary Material

Additional file 1 **Estimates (and standard errors) of functional connectivity between striatal regions-of-interest and frontal cortical regions for control, medicated depressed, and non-medicated depressed participants**. Frontal cortical region clusters were identified in the between-groups (control vs depressed) analyses; for each participant, Fisher transformed correlation coefficients were averaged across all voxels falling within a given cluster. In no analysis did medicated and non-medicated depressed participants differ significantly from each other. CTL = control; MDD = major depressive disorder; PFC = prefrontal cortex; sACC = subgenual anterior cingulate cortex. Click here for file

Additional file 2 **Functional connectivity with striatal seeds: control (CTL) group**. Voxel-wise *p *< 0.005, minimum cluster size = 26 voxels. ACC = anterior cingulate cortex; DC = dorsal caudate; DCP = dorsal caudal putamen; PFC = prefrontal cortex; VRP = ventral rostral putamen; VS = ventral striatum. Click here for file

Additional file 3 **Functional connectivity with striatal seeds: major depressive disorder (MDD) group**. Voxel-wise *p *< 0.005, minimum cluster size = 26 voxels. ACC = anterior cingulate cortex; DC = dorsal caudate; DCP = dorsal caudal putamen; PFC = prefrontal cortex; VRP = ventral rostral putamen; VS = ventral striatum. Click here for file
