# Supplementary material for: Frontostriatal functional connectivity in major depressive disorder
Source: Biol Mood Anxiety Disord. 2011 Dec 8;1:11. doi: 10.1186/2045-5380-1-11 (PMC3384258; doi:10.1186/2045-5380-1-11)
Supplement: Additional file 1 — Estimates (and standard errors) of functional connectivity between striatal regions-of-interest and frontal cortical regions for control, medicated depressed, and non-medicated depressed participants. Frontal cortical region clusters were identified in the between-groups (control vs depressed) analyses; for each participant, Fisher transformed correlation coefficients were averaged across all voxels falling within a given cluster. In no analysis did medicated and non-medicated depressed participants differ significantly from each other. CTL = control; MDD = major depressive disorder; PFC = prefrontal cortex; sACC = subgenual anterior cingulate cortex. [file 2045-5380-1-11-S1.PDF]

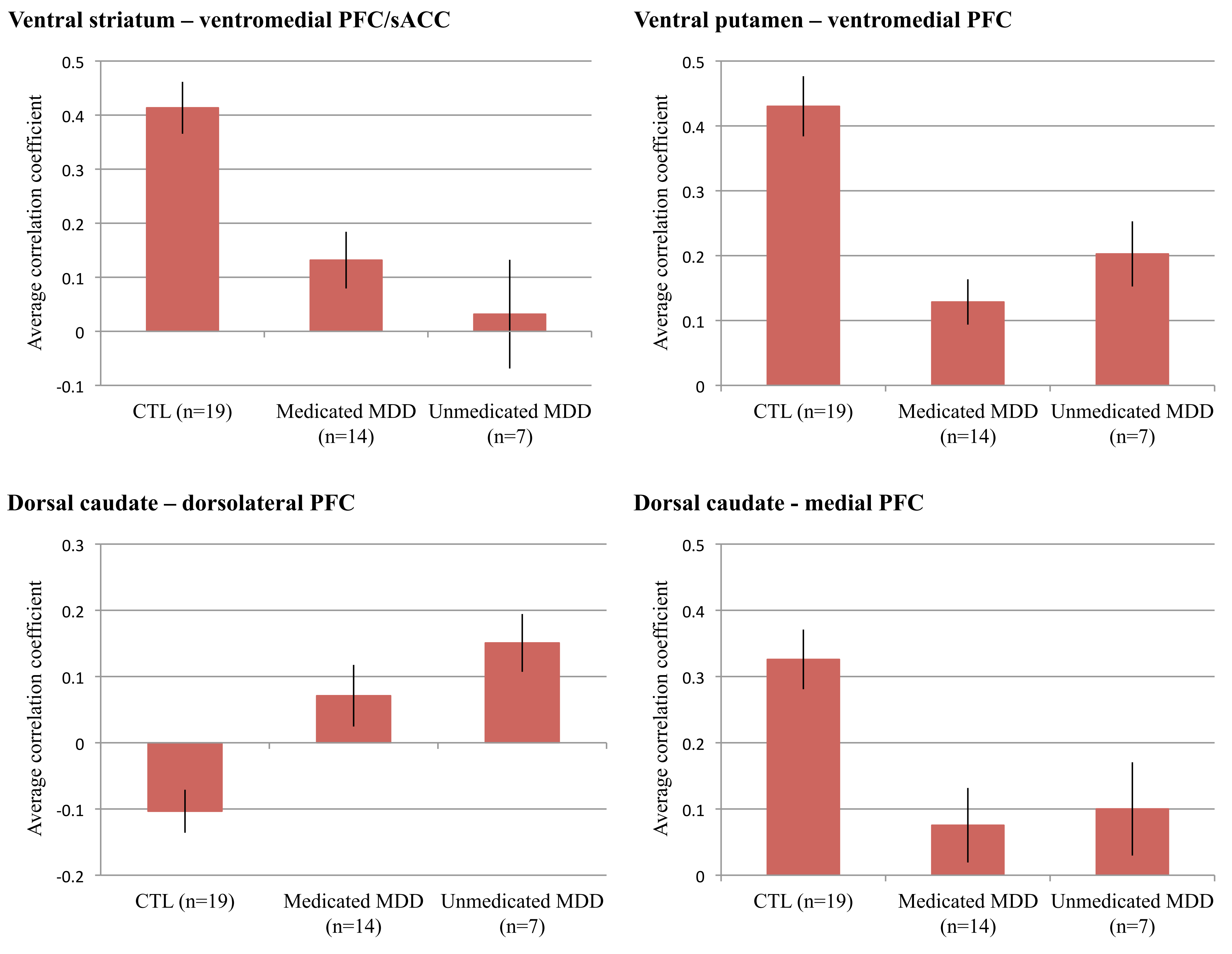
Ventral striatum – ventromedial PFC/sACC
Average correlation coefficient
0.5
0.4
0.3
0.2
0.1
0
-0.1
CTL (n=19)
Medicated MDD (n=14)
Unmedicated MDD (n=7)
Ventral putamen – ventromedial PFC
Average correlation coefficient
0.5
0.4
0.3
0.2
0.1
0
CTL (n=19)
Medicated MDD (n=14)
Unmedicated MDD (n=7)
Dorsal caudate – dorsolateral PFC
Average correlation coefficient
0.3
0.2
0.1
0
-0.1
-0.2
CTL (n=19)
Medicated MDD (n=14)
Unmedicated MDD (n=7)
Dorsal caudate - medial PFC
Average correlation coefficient
0.5
0.4
0.3
0.2
0.1
0
CTL (n=19)
Medicated MDD (n=14)
Unmedicated MDD (n=7)
