# Supplementary material for: Frontostriatal functional connectivity in major depressive disorder
Source: Biol Mood Anxiety Disord. 2011 Dec 8;1:11. doi: 10.1186/2045-5380-1-11 (PMC3384258; doi:10.1186/2045-5380-1-11)
Supplement: Additional file 2 — Functional connectivity with striatal seeds: control (CTL) group. Voxel-wise p < 0.005, minimum cluster size = 26 voxels. ACC = anterior cingulate cortex; DC = dorsal caudate; DCP = dorsal caudal putamen; PFC = prefrontal cortex; VRP = ventral rostral putamen; VS = ventral striatum. [file 2045-5380-1-11-S2.DOC]

**Additional File 2: Functional connectivity with striatal seeds – control (CTL) group**

| Seed | Direction | Region | Peak voxel (Talairach) | | | Cluster size | Peak  t-value |
| --- | --- | --- | --- | --- | --- | --- | --- |
|  |  |  | x | y | z |  |  |
| VS | Positive | Bilateral nucleus accumbens, putamen, caudate, subgenual ACC, medial frontal cortex, thalamus, hippocampus | 14 | 8 | 7 | 4455 | 18.8 |
|  | Negative | R. middle frontal gyrus | 44 | 26 | 35 | 390 | 6.9 |
|  |  | R. middle frontal gyrus | 35 | 50 | 11 | 160 | 6.2 |
|  |  | L. superior frontal gyrus | -40 | 35 | 32 | 277 | 7.5 |
|  |  | L. inferior parietal lobule | -61 | -37 | 35 | 307 | 7.5 |
|  |  | R. supramarginal gyrus, inferior parietal lobule | 35 | -49 | 35 | 161 | 5.2 |
|  |  | L. precuneus | -1 | -64 | 44 | 38 | 4.3 |
|  |  | L. cerebellum | -46 | -70 | -22 | 67 | 4.7 |
|  |  | R. cerebellum | 32 | -28 | -28 | 33 | 4.1 |
|  |  | Sub-gyral | -19 | -22 | 32 | 91 | 5.0 |
|  |  |  |  |  |  |  |  |
| DC | Positive | Bilateral caudate, putamen, thalamus, pregenual ACC, ventromedial PFC, insula | -16 | 17 | 5 | 4800 | 37.7 |
|  |  | R. cerebellum (culmen) | 38 | -49 | -28 | 55 | 4.4 |
|  |  | R. inferior frontal gyrus | 56 | 20 | 11 | 51 | 5.2 |
|  |  | L. ACC, medial frontal cortex | -4 | 20 | 41 | 27 | 4.1 |
|  | Negative | R. precuneus | 2 | -85 | 38 | 145 | 5.1 |
|  |  | Pons | 8 | -19 | -34 | 78 | 5.3 |
|  |  | L. inferior temporal gyrus | -58 | -34 | -28 | 28 | 4.5 |
|  |  | Sub-gyral | -22 | -16 | 38 | 98 | 5.7 |
|  |  | Sub-gyral | 23 | -10 | 38 | 57 | 6.2 |
|  |  |  |  |  |  |  |  |
| VRP | Positive | Bilateral putamen, caudate, insula, subgenual ACC, pregenual ACC, thalamus, hypothalamus | 17 | 8 | -4 | 5206 | 26.7 |
|  |  | Medial frontal gyrus | -1 | 2 | 44 | 56 | 4.5 |
|  | Negative | R. lingual gyrus, fusiform gyrus | 11 | -88 | -7 | 110 | 5.3 |
|  |  | L. fusiform gyrus | -43 | -70 | -19 | 44 | 5.1 |
|  |  | L. lingual gyrus | -7 | -85 | -16 | 37 | 5.1 |
|  |  | L. supramarginal gyrus, inferior parietal lobule | -34 | -52 | 35 | 48 | 3.7 |
|  |  | Sub-gyral | 16 | 14 | 32 | 692 | 3.9 |
|  |  | Sub-gyral | -22 | -13 | 32 | 132 | 8.7 |
|  |  | Sub-gyral | -25 | 14 | 26 | 32 | 4.4 |
|  |  |  |  |  |  |  |  |
| DCP | Positive | Bilateral putamen, caudate, insula, thalamus, medial frontal cortex, superior temporal gyrus, amygdala, postcentral gyrus | -25 | 5 | 5 | 7724 | 26.0 |
|  |  | R. precentral gyrus | 20 | -19 | 53 | 49 | 4.4 |
|  |  | R. cuneus, posterior cingulate cortex | 11 | -61 | 11 | 45 | 4.4 |
|  |  | R. superior temporal gyrus | 59 | -61 | 11 | 45 | 5.2 |
|  | Negative | Bilateral lingual gyrus, inferior occipital gyrus | 8 | -91 | -7 | 470 | 6.9 |
|  |  | R. inferior parietal lobule, superior parietal lobule | 41 | -46 | 47 | 238 | 6.0 |
|  |  | R. precuneus | 2 | -64 | 41 | 204 | 6.0 |
|  |  | L. inferior parietal lobule, superior parietal llobule | -58 | -73 | 29 | 89 | 4.7 |
|  |  | R. inferior temporal gyrus | 56 | -13 | -25 | 50 | 5.1 |
|  |  | R. medial frontal cortex | 11 | 38 | 32 | 27 | 4.6 |
|  |  | Sub-gyral, R. precentral gyrus | 23 | -22 | 26 | 240 | 8.1 |
|  |  | Sub-gyral | -25 | -4 | 26 | 103 | 5.5 |

Notes: VS = ventral striatum; DC = dorsal caudate; VRP = ventral rostral putamen; DCP = dorsal caudal putamen; ACC = anterior cingulate cortex; PFC = prefrontal cortex; voxel-wise p < 0.005, minimum cluster size = 26 voxels
