# Supplementary material for: Frontostriatal functional connectivity in major depressive disorder
Source: Biol Mood Anxiety Disord. 2011 Dec 8;1:11. doi: 10.1186/2045-5380-1-11 (PMC3384258; doi:10.1186/2045-5380-1-11)
Supplement: Additional file 3 — Functional connectivity with striatal seeds: major depressive disorder (MDD) group. Voxel-wise p < 0.005, minimum cluster size = 26 voxels. ACC = anterior cingulate cortex; DC = dorsal caudate; DCP = dorsal caudal putamen; PFC = prefrontal cortex; VRP = ventral rostral putamen; VS = ventral striatum. [file 2045-5380-1-11-S3.DOC]

**Additional File 3: Functional connectivity with striatal seeds – depressed (MDD) group**

| Seed | Direction | Region | Peak voxel (Talairach) | | | Cluster size | Peak  t-value |
| --- | --- | --- | --- | --- | --- | --- | --- |
|  |  |  | x | y | z |  |  |
| VS | Positive | Bilateral nucleus accumbens, putamen, caudate, subgenual ACC, ventromedial PFC, thalamus, parahippocampal gyrus | 11 | 8 | -7 | 5376 | 19.7 |
|  |  | L. middle cingulate cortex | -4 | 8 | 38 | 123 | 4.6 |
|  |  | R. middle temporal gyrus, superior temporal gyrus | 56 | -1 | -16 | 28 | 4.7 |
|  | Negative | R. inferior parietal lobule | 44 | -40 | 41 | 168 | 7.1 |
|  |  | L. supramarginal gyrus, inferior parietal lobule | -55 | -37 | 32 | 39 | 4.6 |
|  |  | L. middle frontal gyrus | -49 | 44 | -4 | 76 | 4.6 |
|  |  | R. middle frontal gyrus | 35 | 44 | 29 | 48 | 4.5 |
|  |  | L. middle temporal gyrus | -55 | -34 | -10 | 28 | 4.2 |
|  |  |  |  |  |  |  |  |
| DC | Positive | Bilateral caudate, putamen, thalamus, dorsal ACC, pregenual ACC, middle frontal gyrus, medial frontal cortex | -13 | 14 | 8 | 4015 | 31.5 |
|  |  | L. middle frontal gyrus | -25 | 14 | 41 | 28 | 4.1 |
|  | Negative | L. precuneus, cuneus | -10 | -82 | 38 | 77 | 5.3 |
|  |  | L. middle occipital gyrus, cuneus | -25 | -76 | 8 | 28 | 4.2 |
|  |  |  |  |  |  |  |  |
| VRP | Positive | Bilateral putamen, caudate, insula, subgenual ACC, dorsal ACC, middle cingulate cortex, inferior frontal cortex, inferior parietal lobule | -19 | 8 | -1 | 5732 | 37.3 |
|  |  | L. middle frontal gyrus | -31 | 41 | 23 | 107 | 6.4 |
|  | Negative | L. precuneus | -4 | -79 | 41 | 51 | 4.4 |
|  |  | L. middle temporal gyrus | -67 | -28 | -16 | 48 | 5.9 |
|  |  |  |  |  |  |  |  |
| DCP | Positive | Bilateral putamen, caudate, thalamus, insula, inferior frontal gyrus, postcentral gyrus, inferior parietal lobule, precentral gyrus | -28 | 2 | 2 | 7406 | 32.8 |
|  |  | Bilateral middle cingulate cortex | -7 | 8 | 35 | 922 | 7.3 |
|  |  | L. middle frontal gyrus | -31 | 41 | 26 | 201 | 10.3 |
|  |  | R. superior frontal gyrus, middle frontal gyrus | 29 | 47 | 23 | 50 | 4.7 |
|  | Negative | L. precuneus, cuneus | -1 | -82 | 44 | 112 | 5.2 |
|  |  | L. precuneus | -13 | -52 | 32 | 45 | 4.8 |
|  |  | L. middle temporal gyrus | -40 | -61 | 26 | 107 | 5.4 |
|  |  | L. middle temporal gyrus | -55 | -19 | -10 | 72 | 5.1 |
|  |  | Bilateral ventromedial PFC | 2 | 44 | -10 | 55 | 5.3 |
|  |  | Bilateral medial frontal pole | -1 | 71 | 17 | 34 | 4.3 |
|  |  | L. cerebellum | -28 | -58 | -16 | 50 | 4.6 |
|  |  | L. cerebellum | -61 | -49 | -25 | 35 | 4.3 |
|  |  | R. cerebellum | 17 | -43 | -19 | 32 | 4.2 |
|  |  | Sub-gyral | 23 | -25 | 29 | 71 | 6.6 |
|  |  | Sub-gyral | -25 | -22 | 29 | 43 | 5.2 |

Notes: VS = ventral striatum; DC = dorsal caudate; VRP = ventral rostral putamen; DCP = dorsal caudal putamen; ACC = anterior cingulate cortex; PFC = prefrontal cortex; voxel-wise p < 0.005, minimum cluster size = 26 voxels
